# Experimental and simulation study on heat transfer characteristics of aluminium alloy piston under transition conditions

**DOI:** 10.1038/s41598-022-13357-0

**Published:** 2022-06-03

**Authors:** Yang Liu, Jilin Lei, Dongfang Wang, Xiwen Deng, Jun Wen, Zhigao Wen

**Affiliations:** 1 Yunnan Key Laboratory of Internal Combustion Engines, Kunming University of Science and Technology, Kunming, 650500 People’s Republic of China; 2 Yunnan Key Laboratory of Plateau Emission of Internal Combustion Engines, Kunming Yunnei Power CO., LTD, Kunming, 650200 People’s Republic of China; 3 Chengdu Galaxy Power CO., LTD, Chengdu, 610505 People’s Republic of China

**Keywords:** Engineering, Mechanical engineering

## Abstract

In order to explore the thermal load change of the diesel engine piston under transitional conditions, and the influence of the position of cooling gallery on the heat transfer characteristics of the piston. An off-road high-pressure common-rail diesel engine is chosen as the research object. The sequence coupling method is used to establish the fluid–solid coupling heat transfer simulation model of the piston-gallery under the transition conditions of cold start, urgent acceleration and rapid deceleration. The Pareto optimization algorithm is introduced to optimize the position of the cooling gallery to reduce the maximum temperature and maximum thermal stress of the piston. The results show that the maximum temperature of the piston can be reduced by reducing the distance between the cooling gallery and the throat area under the maximum torque condition, and that the maximum thermal stress of the piston can be reduced by reducing the distance between the cooling gallery and the throat area or by increasing the distance between the cooling gallery and the ring area. Compared with the original design, the maximum temperature of Design A decreases by 1.28 °C while the maximum thermal stress decreases by 2.07 MPa. The maximum temperature and maximum thermal stress of Design B decreases by 0.22 °C and 0.5 MPa, respectively. The maximum thermal stress of Design C decreases by 2.67 MPa when the maximum temperature increases by 1.15 °C. The maximum change in temperature of the three typical designs and the original design of the piston throat under cold start, urgent acceleration and rapid deceleration conditions reached 207.29 °C, 136.78 °C and 9.89 °C, and the maximum change of thermal stress reached 8.62 MPa, 20.43 MPa, 4.08 MPa, respectively. The maximum change in temperature of the piston first ring groove under cold start, urgent acceleration and rapid deceleration conditions reached 172.00 °C, 83.52 °C and 7.36 °C, and the maximum change in thermal stress reached 22.96 MPa, 43.10 MPa, 5.72 MPa, respectively. The conclusions obtained can provide boundary conditions for further study of the thermal load change law of the same type of pistons, and also provide a theoretical basis for diesel engine piston structure optimization and the performance improvement.

## Introduction

Diesel engines continue to develop in the direction of high-strength, lightweight, compactness, and low emissions. The amount of heat released per unit time has increased significantly, the temperature and pressure in the cylinder have risen sharply, and the environment in the cylinder is getting worse^1–4^. As one of the most important components in the engine, the working state of the piston in the cylinder is the most complex. The top surface of the piston is simultaneously subjected to the dual effects of high-temperature gas and the pressure in the cylinder, and the thermal shock it bears is getting higher and higher. Unreasonable design of piston structure will lead to piston failure during engine operation, among which are hot cracks, metal melting, and ablation^5,6^. Therefore, the analysis and understanding of the heat transfer characteristics of the piston under the transient conditions is crucial to the design of the piston with high reliability and high durability.

Researchers have conducted a lot of research on piston related fields, among which the thermal load research of the piston is mainly concentrated on the steady-state working condition under the calibrated power condition and the maximum torque condition. However, only considering the thermal load of the piston under steady operating conditions cannot fully reflect the actual change of the thermal load of the piston during the operation of the engine. With the further development of temperature sensor technology, the transient temperature test of the piston is carried out. Researchers have conducted a lot of explorations on the transient temperature field test of piston^7–17^, such as the application of thermocouple temperature measurement technology, storage temperature measurement technology and infrared temperature measurement technology in the piston temperature test, which makes the piston temperature field test move from the steady state test to the transient test. And it is found that the mechanical load and thermal load of the engine under variable operating conditions is relatively large, exceeding 10–25%^18^ of it’s the condition of calibrated power, which has a great impact on the reliability and life of the engine^19^.

On the basis of experimental tests, numerical simulation technology has been widely used in the calculation of piston transient thermal load. In 1947, Donea^20^ was the first to compute and analyse the nonstationary temperature field using the Galerkin format based on the weighted residual method, and then Wilson and Nickell^21^ computed the nonstationary temperature field using the format of central difference based on the variational principle. In 1987, Chen et al.^22^ established a piston-cylinder coupled heat transfer model by considering the nonlinear heat transfer of gas convection and heat radiation in the cylinder and the transient temperature field and heat flux distribution of the piston was predicted and analysed. Subsequently, this piston-cylinder coupled heat transfer method is widely used^23–26^. In 1990, Prasad et al.^27^ used the finite difference method to study the transient temperature response of the piston top surface, and on this basis, studied the influence of an oxide coating on the temperature field of the piston. In 1991, Li et al.^28^ proposed a thermal boundary condition loading method for finite element calculation of piston under transient conditions based on test results, which provided a basis for calculation of piston temperature field under variable conditions. In 2006, Song et al.^29^ used spatial shaping of the laser beam to induce changes in the piston temperature field to study the transient heat load of the piston under steady-state and variable operating conditions, and used a high-definition CCD camera device to monitor the piston surface cracks. In 2013, Liu et al.^30^ predictively analysed the exponential increase in piston temperature under diesel engine start-up conditions with a large quasi-static thermal stress, but did not investigate the changes in the piston transient temperature field during urgent acceleration and deceleration. In 2016, Peng et al.^31^ used storage piston temperature testing technology to study the effect of engine speed and torque on piston temperature. In 2017, Liu et al.^32^ investigated the transient temperature field distribution and thermal stress distribution of the piston under calibration conditions and showed that temperature fluctuations within 2 mm of the top surface of the piston produce large thermal stress fluctuations.

In recent years, in order to reduce the thermal load of the piston and maintain a high strength of the piston, the oscillatory heat transfer in the cooling gallery, as an efficient way of enhancing the heat transfer, has been widely used in high thermal load engine pistons. The optimisation of the heat transfer characteristics of the cooling gallery is primarily aimed at optimising the temperature field of the piston, which is indexed to include the maximum temperature of the piston and maximum temperature gradient of the piston. Thermal stress is also a function of the temperature gradient and the magnitude of the thermal stress also characterises the service life of the piston, which can be increased by reducing the thermal stress. Study has shown that the cooling gallery can reduce the temperature of the top surface of the piston by about 40 °C, taking away about 40% to 60% of the heat dissipated by the entire piston^33^. The cooling gallery reduces the operating temperature of the piston while also producing a large change in the operating temperature gradient of the piston, resulting in a large thermal stress. Unreasonable design of the cooling gallery will lead to thermal fatigue failure of the piston. Therefore, the rational design of the location of the cooling gallery is the key to reducing the thermal load on the piston and ensuring its operational reliability.

Based on the above reasons, this paper takes the aluminium alloy piston of an off-road high-pressure common-rail diesel engine as the research object, and uses the method of combining experimental test and simulation calculation to study the variation law of the transient temperature field and thermal stress of the piston under the transition conditions of cold start, urgent acceleration and rapid deceleration. And the Pareto optimization algorithm is introduced to optimize the position of the cooling gallery. The purpose of this study is to develop the optimization methodology for the cooling gallery to decrease the maximum temperature and maximum thermal stress of the piston.

## Methodology

### Research subject

The research subject is an off-road high-pressure common-rail diesel engine, in which the aluminium alloy piston is cooled by an internal cooling gallery and the piston chamber shape is an indented ω type. The relevant parameters for the diesel engine are listed in Table 1.

**Table 1:** Main parameters of the engine.

Parameter	Value
Engine displacement (L)	3.92
Calibrated power (kW)	125
Calibrated speed (r/min)	2600
Maximum torque (N m)	600
Maximum torque speed (r/min)	1300 ~ 1900
Compression ratio	17.2
Bore × Stroke (mm)	102 × 120

### Experiment program

The piston is located within the engine, surrounded by the cylinder liner and the body. The piston head is impacted by high-temperature gas and is in a state of high speed reciprocating motion. Therefore, the accurate measurement of the transient temperature at the top of the piston head has been challenging while studying transient thermal loads. In this paper, a TT-K-30 thermocouple and lead transmission system is used to measure the temperature field of the diesel engine piston under starting condition, urgent acceleration condition and rapid deceleration condition. TT for 46Perfluoroalkoxy material, which can bend 200,000 times at 82 °C, can meet the requirement of reciprocating bending under engine operation. K is a K-type thermocouple, which is a kind of base metal thermocouple that can take high temperature, and is now widely used as a temperature measuring element in industrial automation control systems. The 30 is the wire gauge of 30, and the single-strand size of the thermocouple after being wrapped with 46 Perfluoroalkoxy material is 0.6 mm × 1.0 mm. The test temperature range of the thermocouple is 0–1250 °C, and the thermocouple tolerance is 1.1 °C or 0.4%. The thermocouple adopts OMEGA non-impurity solder joint technology to weld into spherical solder joints, thus ensuring the accuracy of measurement, and the bare type can improve the response speed, and its response time is less than 5 ms. Before the piston temperature field test, the thermocouple sensor used was pre-calibrated from 0 to 400 °C. Technical parameters of thermocouple sensor are listed in Table 2.

**Table 2:** Technical parameters of the thermocouple sensor.

Parameter	Value
Output voltage (V)	0 ~ 4
Calibration range (°C)	0 ~ + 400
Seebeck coefficient (μV/°C)	40
Thermocouple response time (ms)	≤ 5
Output voltage error (%)	≤ ± 0.4
Working environment temperature of the cold end (°C)	0 ~ 35
Working environment humidity of the cold end (%RH)	30 ~ 90
Preheating time (min)	≤ 15
Additional error of ambient temperature (°C)	≤ ± 0.3
Continuous working time of the instrument (h)	4

**Figure 1:**
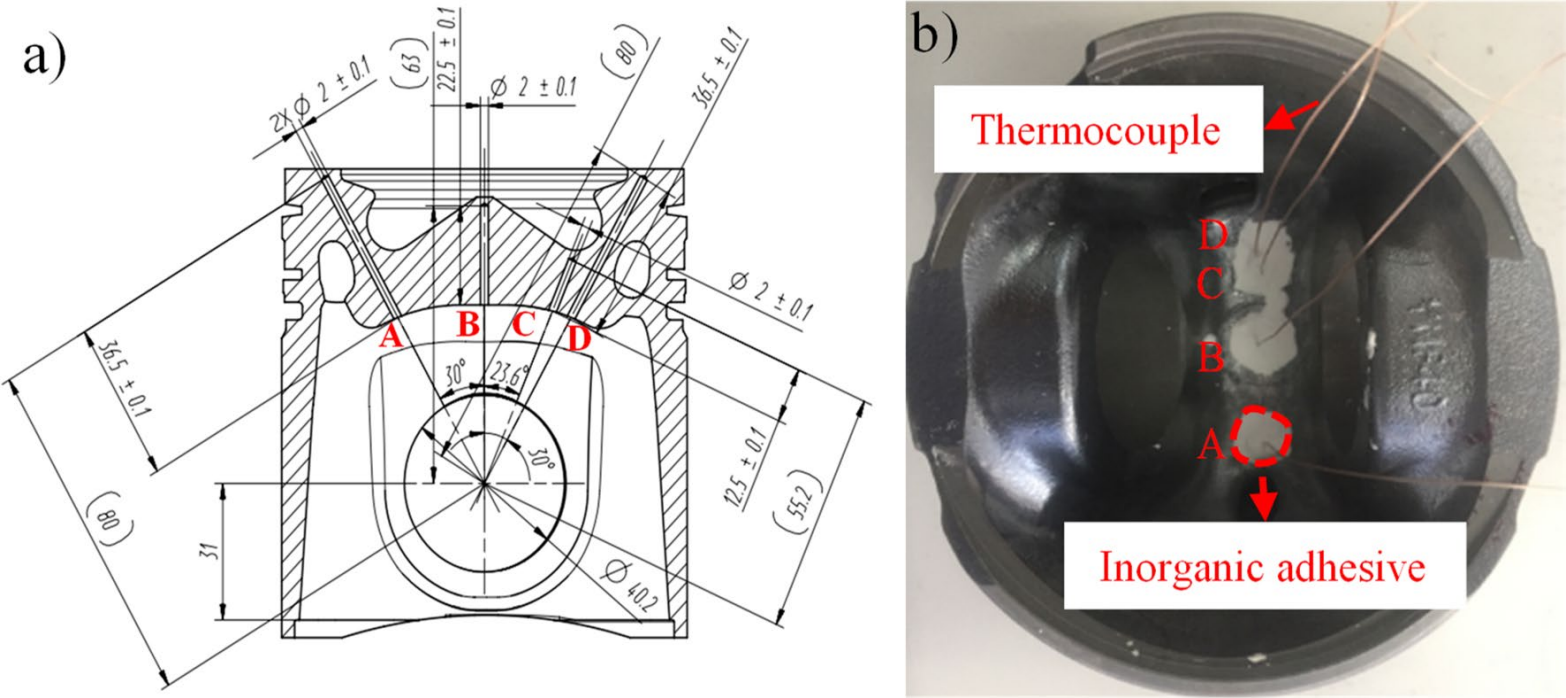
Sketch map of piston measuring points of (**a**) cutaway view and (**b**) physical view.

A single piston is arranged with four measurement points located at the centre of the piston combustion chamber, the bottom of the piston chamber, and top surface of the piston. According to the position of the measuring point, a hole was drilled from the inner cavity of the piston to a position 2 mm below the top surface of piston. The measuring point of the thermocouple was placed into the bottom of the hole and the copper oxide inorganic glue was poured to secure, as shown in Fig. 1. During the drilling process, the drilling angle and depth should be monitored closely to avoid deviations in the drilling position, and the internal cooling gallery should be penetrated. After the thermocouple sensor is installed, the thermocouples are numbered to facilitate the recording of the measurement results to ensure the integrity of the sensor using a voltmeter.

**Figure 2:**
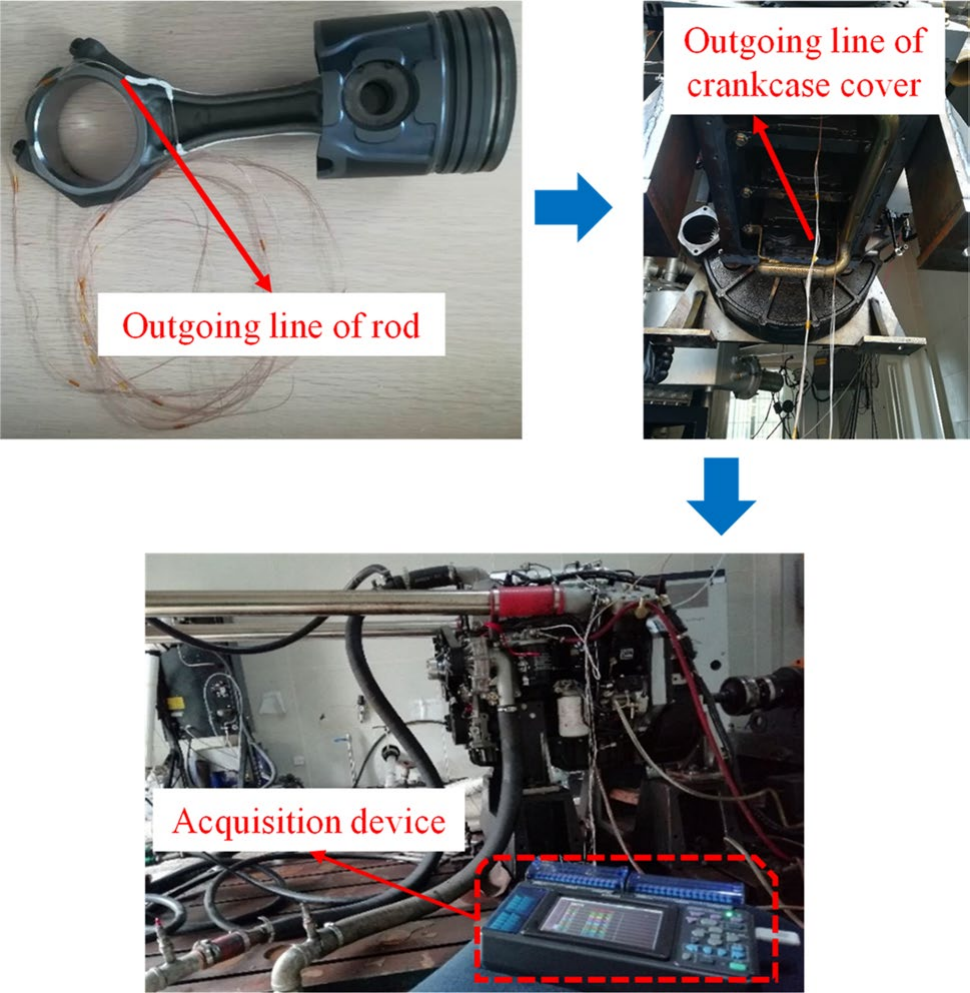
Lead physical diagram of thermocouple wire.

Considering the process of the short test, no auxiliary mechanism is introduced in the lead process; only slots are made on either side of the connecting rod, as shown in Fig. 2. The thermocouple wire is buried in the connecting rod groove, and poured the copper oxide inorganic glue to secure.

The test is carried out according to the test method of cold start process, urgent acceleration process and rapid deceleration process specified in the Chinese standard ‘Automobile engine reliability test method: GB/T 19055-2003’. As listed in Table 3, the transition condition as follows is tested three times after shutdown and cooling.

**Table 3:** Test and test conditions.

Condition	Parameters
Cold start process	0 ~ Idle speed condition (speed: 800 r/min, fuel injection timing: 5.7°CAbTDC (Degree Crank Angle before Top Dead Centre), fuel injection pressure: 88 MPa, and coolant temperature: 70 °C)
Urgent acceleration process	Idle speed condition (800 r/min) ~ Calibrated speed condition (2400 r/min, fuel injection timing: 10.1°CAbTDC, fuel injection pressure: 133 MPa, and coolant temperature: 74.3 °C)
Rapid deceleration process	Calibrated speed condition (2400 r/min) ~ Maximum torque speed condition (1600 r/min, fuel injection timing: 16°CAbTDC, fuel injection pressure: 145 MPa, and coolant temperature: 79.9 °C)

### Simulation model setup

This chapter involved.

### Grid model

In this paper, HYPERMESH software is used for finite element meshing of the model. In the process of finite element meshing, the top surface of the piston, the unloading groove and the top surface of the piston inner cavity are refined. The average mesh size of the piston model is 1.6 mm. In order to improve the efficiency of calculation while ensuring the accuracy, the chamfering and rounding of the piston skirt and the piston cavity below 3 mm are simplified, but the original geometric features of the piston are retained at the piston top surface position without simplification, and the overall calculation grid number of the piston is 381,754.

### Material characteristics parameters

The piston body material is a silicon aluminium alloy (with a small amount of magnesium), and the piston ring material is austenitic wear-resistant cast iron. The thermal physical parameters of the silicon aluminium alloy materials at different temperatures are listed in Table 4^34^. The specific thermophysical characteristic parameters of the piston ring, pin and rod are listed in Table 5^35^.

**Table 4:** Thermophysical parameters of the piston body material at different temperatures.

Temperature (°C)	Thermal conductivity (W m^−2^ K^−1^)	Density (kg m^−3^)	Specific heat capacity (J kg^−1^ K^−1^)	Linear expansion rate (10^−6^ m m^−1^ K^−1^)
20	130	2810	900	19.2
100	134	2805	920	19.2
150	136	2798	942	19.8
200	139	2791	968	20.5
250	142	2785	973	20.8
300	143	2777	985	21.1
350	145	2773	992	21.3
400	146	2767	1007	21.5

**Table 5:** Thermophysical parameters of the piston ring, pin, and rod.

Part	Thermal conductivity (W m^−2^ K^−1^)	Density (kg m^−3^)	Specific heat capacity (J kg^−1^ K^−1^)	Linear expansion rate (10^−6^ m m^−1^ K^−1^)
Piston ring	44	7300	470	1.80
Pin	50.66	7830	510	1.37
Rod	50.66	7830	510	13.65

### Thermal boundary conditions

**Figure 3:**
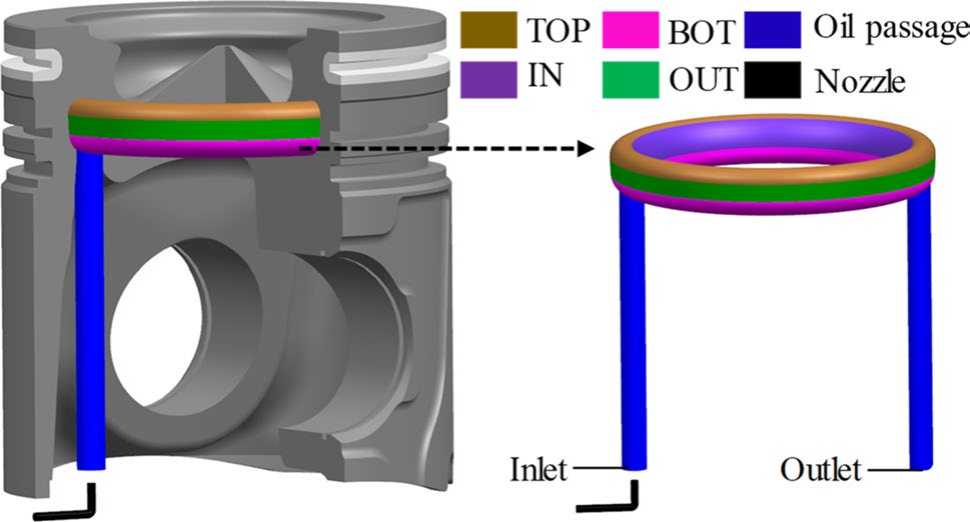
The geometry of the piston gallery and the name of each boundary.

Accurate thermal boundary conditions are the basis for studying the temperature field and thermal load of the piston, and are also the key factors that determine the accuracy of the model's calculations. In this study, the piston studied has an inner cooling gallery, so when determining the thermal boundary conditions in the wall area of the cooling gallery, the oscillating cooling flow and heat transfer of the oil in the cooling gallery should be calculated and analysed by CFD (Computational Fluid Dynamics).The thermal boundary condition of the cooling gallery wall is to map the average temperature and average convective heat transfer coefficient calculated from each area to the piston cooling gallery wall after establishing the oscillating cooling flow and heat transfer simulation model^36^ of the oil in the cooling gallery. The simplified geometry of the cooling gallery and the name of each boundary are shown in Fig. 3. The boundaries were divided into the oil inlet, the air inlet, the walls, the outlet, and the inlet and outlet passages of the cooling gallery. For convenience in description, the walls of the cooling gallery were subdivided into a TOP region an OUT region, an IN region, and a BOT region. The TOP region is located close to the piston crown, the BOT region is located close to the piston chamber. The OUT region is a portion wall of the gallery near the piston ring groove. The IN region is a portion wall of the gallery near the centre of the piston chamber.

**Figure 4:**
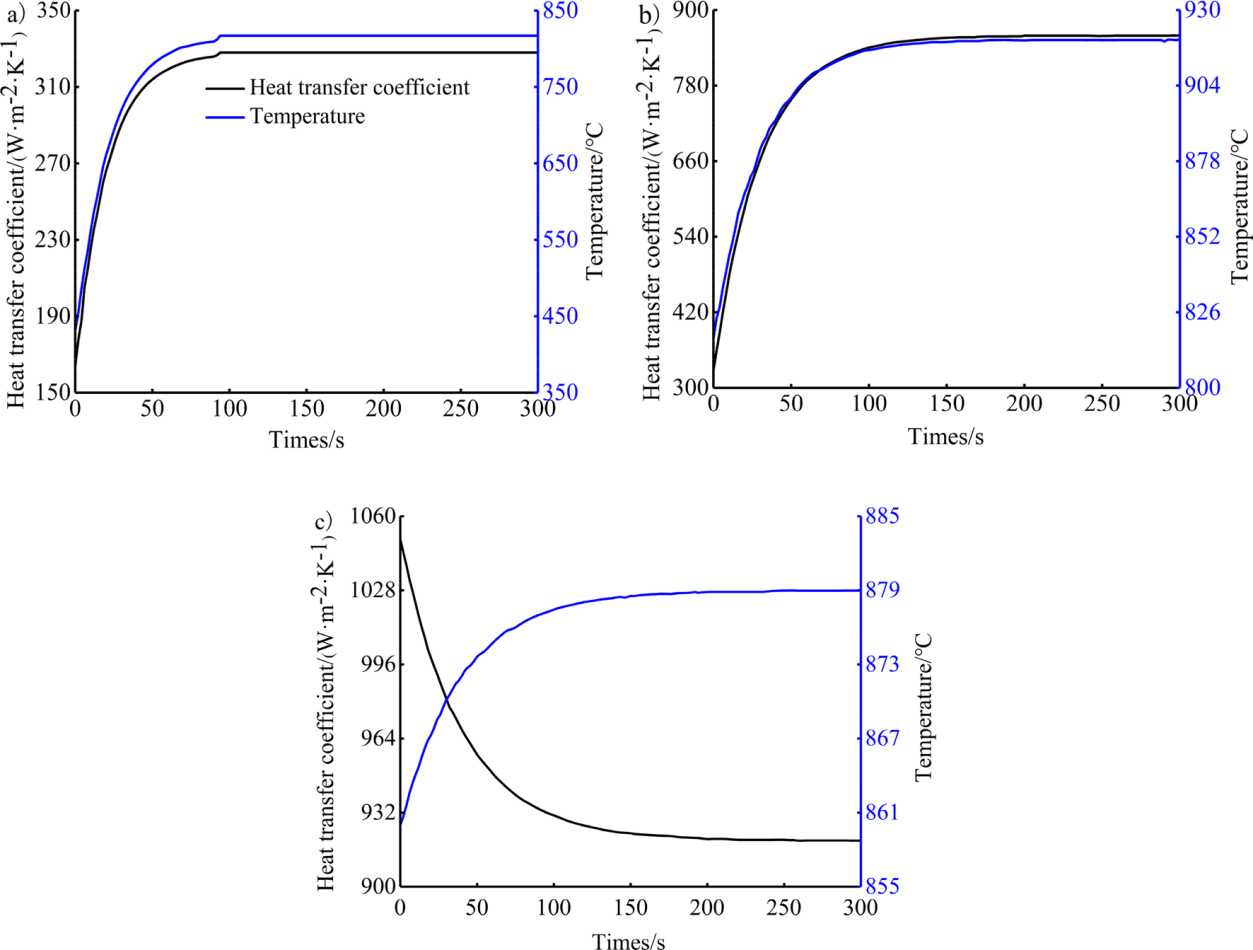
Thermal boundary condition of piston top surface under the (**a**) cold start condition, (**b**) urgent acceleration condition and (**c**) rapid deceleration condition.

In this study, the third type of thermal boundary conditions of the piston top surface and the top land are loaded with time-varying under transient conditions. The thermal boundary conditions of the top surface of the piston and the top land under the transition conditions are loaded using the Eq. (1) ^23,28^. The variation law of the temperature field of the piston under the transition conditions is obtained through experimental tests, and the thermal boundary conditions of the piston under the transition conditions are obtained by assuming that the thermal boundary conditions of the piston are consistent with the variation law of the temperature field of the piston. In the sudden change of engine conditions, the thermal state of the gas in the engine cylinder will produce phenomenon, that is, the boundary parameter of the piston fire surface will suddenly change value at the initial time, the study shows that this sudden change is 1/2 of the value of the whole process parameter change^28^, and then the piston thermal boundary parameter change according to Eq. (1).

1 $$ \left. {\begin{array}{*{20}c}    {\alpha \left( t \right) = \alpha _{1}  + \left( {\alpha _{2}  - \alpha _{1} } \right) \times \left( {1 - e^{{ - \frac{t}{{t_{0} }}}} } \right)}  \\    {T\left( t \right) = T_{1}  + \left( {T_{2}  - T_{1} } \right) \times \left( {1 - e^{{ - \frac{t}{{t_{0} }}}} } \right)}  \\   \end{array} } \right\} $$

where *a*_*1*_ is convective heat transfer coefficient of the starting steady state. *T*_*1*_ is the initial stable state temperature. The process of cold start begins at 25 °C at room temperature. In the urgent acceleration process, the top surface temperature measured at idle condition as the initial temperature, and the rapid deceleration process takes the piston top surface temperature measured at stable condition point as the initial temperature. *a*_*2*_ is convective heat transfer coefficient of the final steady state. *T*_*2*_ is temperature of the final steady state. The *a*_*1*_, *T*_*1*_, *a*_*2*_, and *T*_*2*_ can be obtained from the calculation of the steady-state temperature field. The three aforementioned transition conditions correspond to four steady states: (1) room temperature state before starting, (2) idle speed condition, (3) calibrated power condition and (4) maximum torque condition. *t* is time. *t*_*0*_ is the time constant (i.e., the time corresponding to 63.2% of the total value of the temperature change), which can be read from the measured temperature change curve. According to the test results, it is 20 s under cold start conditions, 30 s under urgent acceleration conditions, and 40 s under rapid deceleration conditions. From these, the thermal boundary loading curve of the piston top surface during cold start condition, urgent acceleration condition and rapid deceleration condition can be obtained, as shown in Fig. 4. The remaining areas of the piston are treated according to the steady-state third type of thermal boundary conditions^37,38^.

### Simulation process

**Figure 5:**
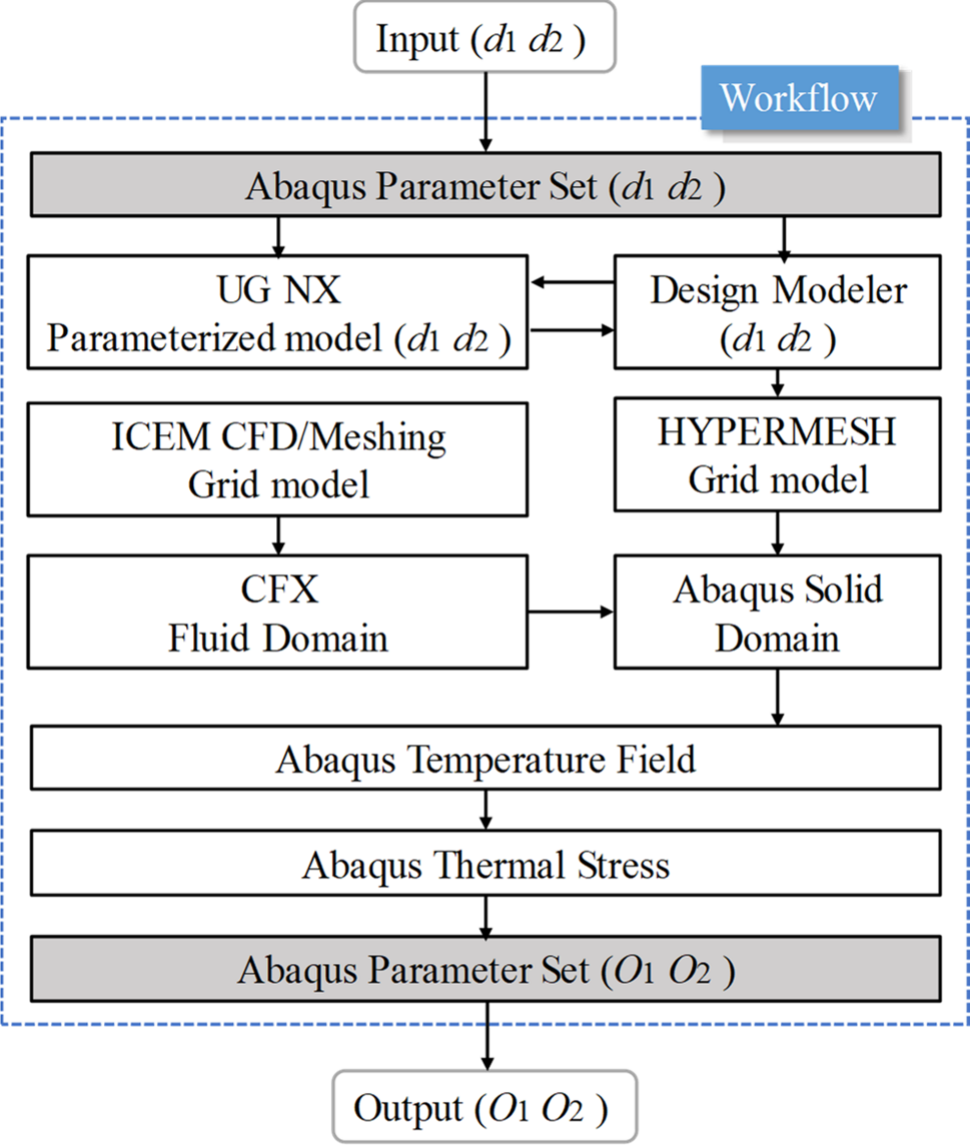
Simulation analysis process.

The fluid–solid coupling between the cooling gallery and the piston adopts the sequential coupling method. The fluid domain calculation model of the cooling gallery is divided into the fluid grid using the ICEM grid software under the ANSYS/Workbench18.0 platform, and the divided fluid grid is imported into the CFX software for calculation. The solid domain of the piston is divided by HYPERMESH grid software, and the divided solid grid is imported into ABAQUS6.13 software for calculation. At this time, the boundary conditions of the cooling gallery use the average temperature and average convective heat transfer coefficient of each boundary of the cooling gallery calculated by CFX software, and finally obtain the temperature field and thermal stress distribution of the piston. To facilitate the subsequent optimization calculation and analysis, the structure of the cooling gallery is parameterized using the software UG NX9.0. The simulation analysis process is shown in Fig. 5.

### Simulation model validation

**Figure 6:**
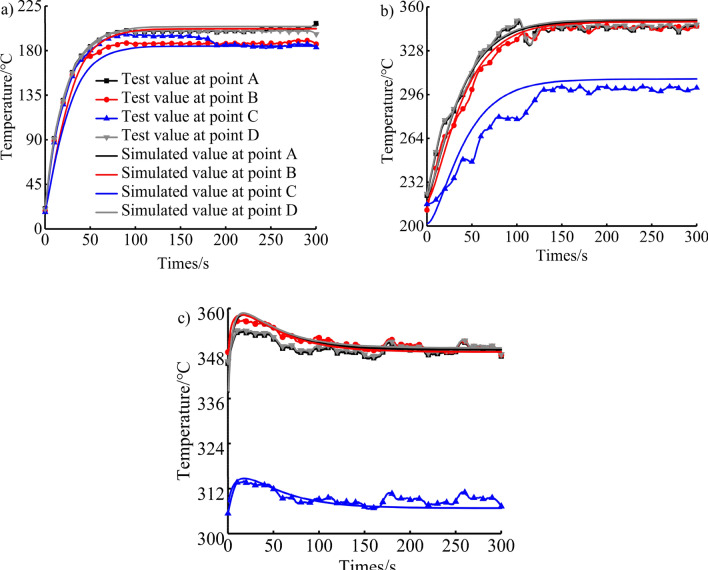
Comparison of test results and simulation results under the (**a**) cold start condition, (**b**) urgent acceleration condition and (**c**) rapid deceleration condition.

Figure 6 shows the temperature simulation results of each measuring point of the piston under the transition conditions of cold start, urgent acceleration and rapid deceleration compared with the test results. The variation trend of the temperature test of the four points on the top surface of the piston is basically consistent with that of the simulation calculation, and the errors are all within 5%, indicating that the finite element simulation model of the piston temperature field under the condition is more accurate. The measuring points A and D are the top surface of the piston, and the measuring point B is the convex point in the centre of the piston chamber. The three measuring points are all in the high temperature area of the combustion flame and therefore have high temperatures. The measuring point C is located at the bottom of the piston chamber and is close to the cooling gallery, so the temperature value is the smallest, which conforms to the law of the piston temperature field. And it can be seen that the highest temperature of the measuring point on the piston surface appears under the maximum torque condition. Table 6 shows the comparison between the average value of simulated calculation of each measuring point of the piston and the average value of experimental test under the maximum torque condition. The maximum error does not exceed 1.40%, and the average error does not exceed 0.81%. Therefore, it is used as the calculation condition for the optimization of the position of the cooling gallery in the next step.

**Table 6:** Comparison of test results and simulation results for maximum torque condition.

Measuring point	Test value (°C)	Simulated value (°C)	Relative error (%)
A	336	340.70	1.40
B	331	333.85	0.86
C	307	306.72	0.09
D	332	329.03	0.89
Average	326.5	327.575	0.81

### Optimization design

This chapter involved.

### Variables design

**Figure 7:**
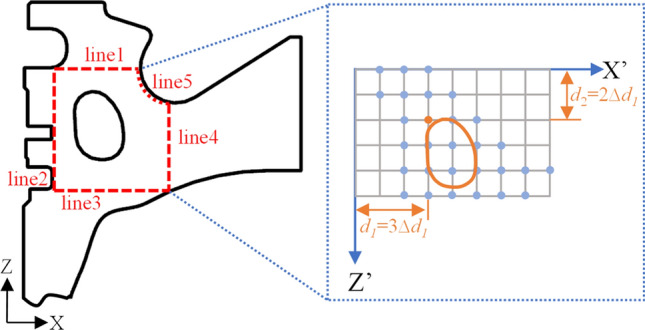
The structure and position parameters of the cooling gallery.

The piston combustion chamber studied is a necked ω-type offset combustion chamber. In order to facilitate the parameterized study of the position of the cooling gallery, the range of limits of the position of cooling gallery was defined during modelling, as shown in Fig. 7. In the figure, line 1 to line 5 are the boundaries of the optimized feasible region. Line 1 is a horizontal line segment, located in the first ring groove of the piston. Line 2 is a vertical line segment, located at the boundary of the piston. Line 3 is a horizontal line segment, located in the third ring groove of the piston. Line 4 is a vertical line segment passing through the centre point of the bottom of the piston chamber. Line 5 is an arc, the centre of which is the same as the centre of the bottom of the piston chamber. The *d*_*1*_ and the *d*_*2*_ are design variables of the cooling gallery in piston. Create X’ and Z’ local coordinate systems based on line 1 and line 2, *d*_*1*_ is the shortest distance between the cooling gallery and line 1; *d*_*2*_ is the shortest distance between the cooling gallery and line 2. On the premise that the shape of the cooling gallery remains unchanged, and to ensure that the position of the cooling gallery determined by the design variables is meaningful, 29 sets of plans are designed with *∆d*_*1*_ = 1.79 mm and *∆d*_*2*_ = 1.71 mm.

### SVR model

**Figure 8:**
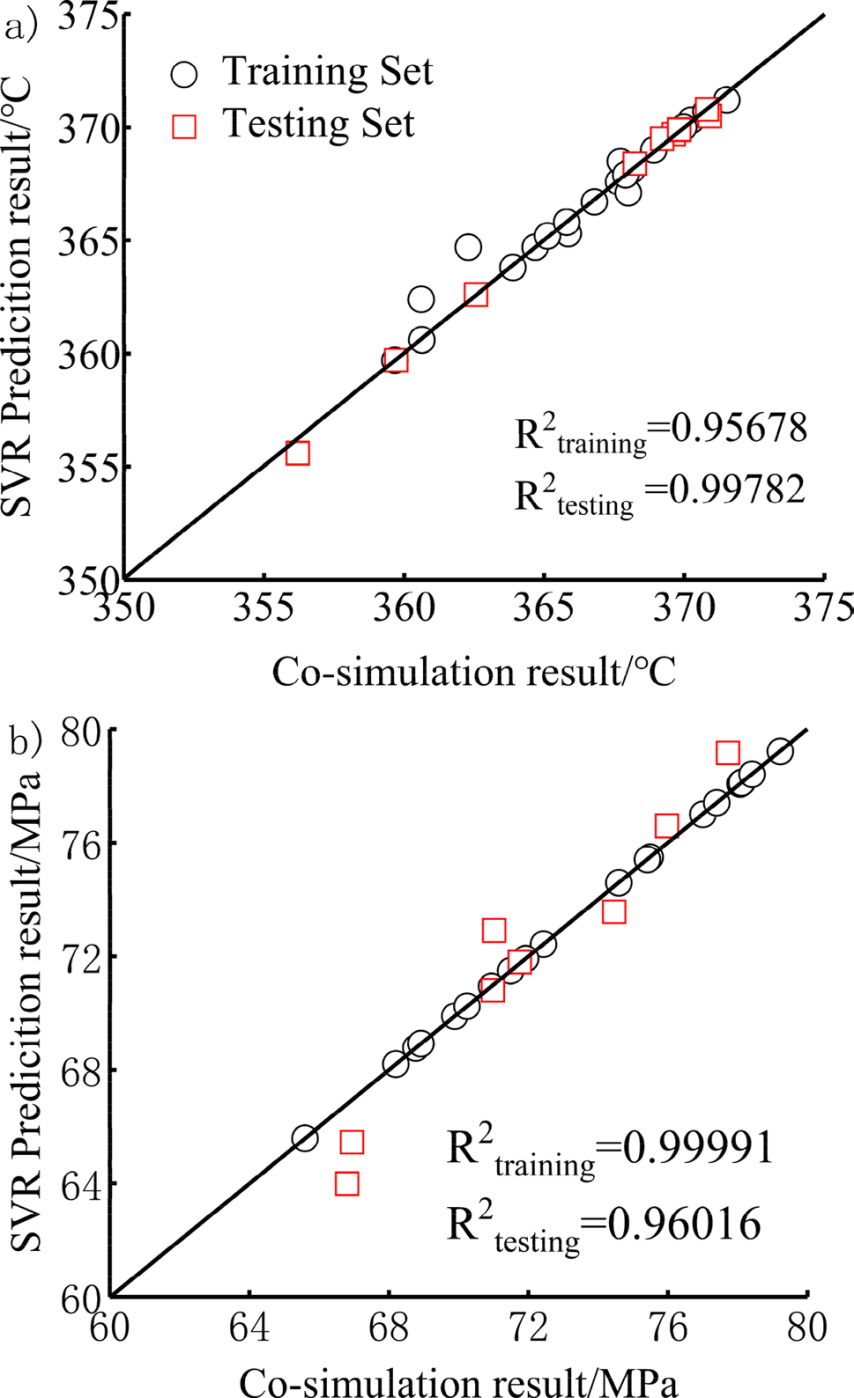
Regression model of (**a**) maximum temperature of piston and (**b**) maximum thermal stress of piston.

Support vector machine (SVM)^39^ is a machine learning method based on statistical learning theory (STL). In order to use SVM to deal with regression fitting problems, Vapinik et al.^40^, based on the research of SVM classification, introduced a Ɛ insensitive loss function to obtain a regression support vector machine (SVR). Combining the above 29 sets of plans plus the original plan, a total of 30 sets of sample points is randomly selected 21 sample points as the training set, and the remaining 9 sample points are used as the test set. This paper is a multi-objective optimization of two functional objectives. Therefore, the support vector machine regression model is two-dimensional. The maximum temperature regression model of the support vector machine and the maximum thermal stress regression model of the support vector machine under the maximum torque condition is established. The model is shown in Eq. (2). The comparison between SVR model prediction results and coupled heat transfer simulation prediction results is shown in Fig. 8. The coefficient of determination R2 of the training set and test set of the two models is above 0.95, indicating that the model has high accuracy and can be used for multi-objective optimization.

2 $$ \begin{aligned} & T = SVR_{T} = \left( {d_{1} ,d_{2} } \right) \\ & S = SVR_{s} = \left( {d_{1} ,d_{2} } \right) \\ \end{aligned} $$

### Multi-objective optimization

The constraint conditions for the optimization of the position of the cooling gallery are mainly to avoid the cross section of the cooling gallery and the piston section from being too close, and to ensure the prevention of excessive temperature gradient and sufficient piston strength. The mathematical description of multi-objective optimization and its related constraints are shown in Eq. (3).

3 $$ \begin{aligned} & \min f_{1} = \min \left[ {\max \left( S \right)} \right] \\ & \min f_{2} = \min \left[ {\max \left( T \right)} \right] \\ & s.t.\left\{ \begin{gathered} 2 \le d_{1} \le 14 \hfill \\ 2 \le d_{2} \le 9 \hfill \\ \end{gathered} \right. \\ \end{aligned} $$

**Figure 9:**
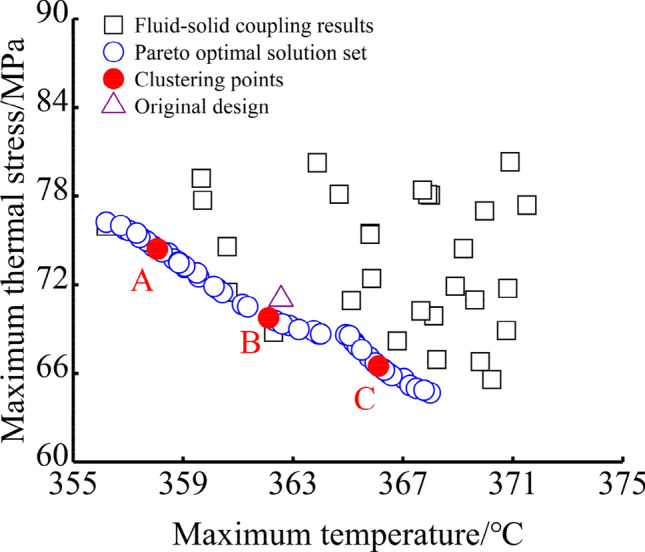
Distribution of clustering points in Pareto optimal solution set.

NSGA-II evolutionary algorithm combined with constraint domination principle is used to find Pareto optimal solution set. The crossover rate is 0.8, the mutation rate is 0.05, the maximum generation of evolution is 100 generations, the generation of stop is 100 generations, and the fitness function value deviation is 1 × 10^–5^. When the fitness function deviation or any condition of the maximum generation of evolution is satisfied, the evolution is terminated and the Pareto optimal solution set is obtained. To analyse the effectiveness of multi-objective optimization, k-means algorithm is used to extract clustering points. In this paper, this algorithm is mainly used to find typical solutions in the Pareto optimal solution set to analyse the effectiveness of multi-objective optimization. By selecting k = 3, 3 clustering points will be found, as shown in Fig. 9. It can be seen from the figure that the three clustering points are evenly distributed in the Pareto optimal solution set. For convenience of the description, the three points are named Design A, Design B, and Design C. The design goal of the cooling gallery represented by Design A is lower maximum piston temperature and higher thermal stress. The design goal of the cooling gallery represented by Design C is a higher maximum piston temperature and lower thermal stress. And design B is between design A and design C. For different design goals, different weights of target need to be selected to obtain a suitable solution for the position of the cooling gallery.

## Results and discussions

### Analysis of heat transfer characteristics of piston under maximum torque condition

**Figure 10:**
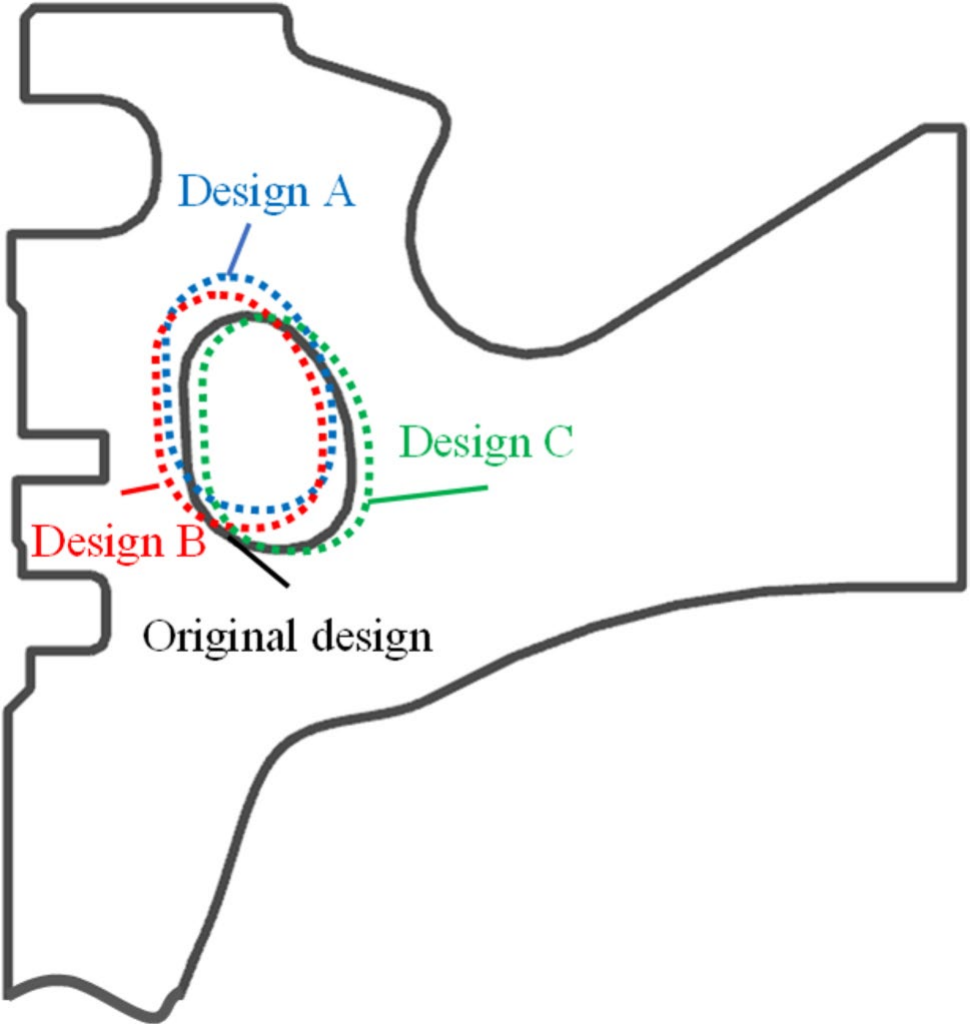
The three optimal gallery sections.

**Figure 11:**
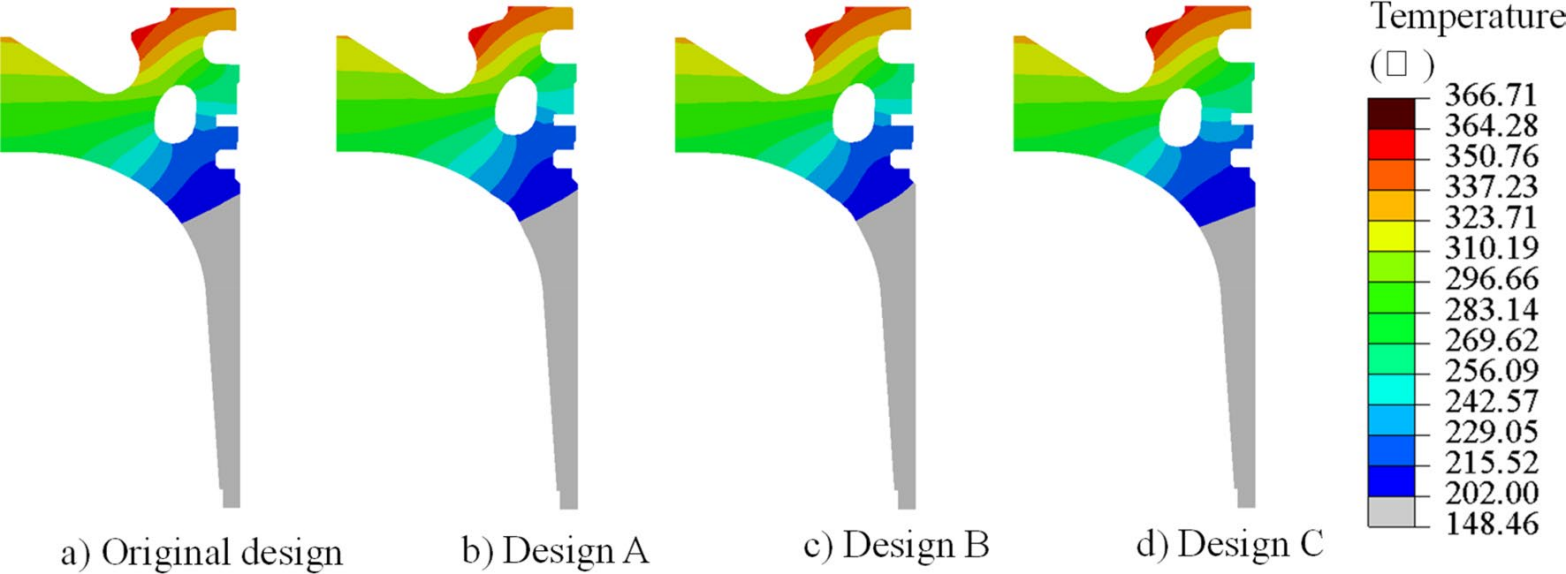
Temperature comparison of original design and cluster designs.

Table 7 lists the variable values and the objective function values of the three clustered points and the original design. Figure 11 illustrates the three optimal cross section of the cooling gallery. According to the variable values in Table 7 and Fig. 10, the cooling gallery of Design A is closer to the piston throat area, which can take away more heat in the throat area, so that the maximum temperature of the piston can be reduced by 1.28 °C. Because the distance between the cooling gallery in Design A and the ring area is between Design B and the original design, the maximum thermal stress of the piston can be reduced by 2.07 MPa. The cooling gallery of Design B is closer to the piston ring area, and the wall thickness of this design is the smallest, which will still generate greater thermal stress, so the maximum thermal stress is only reduced by 0.5 MPa compared to the original design. The distance between the cooling gallery and the throat of Design B is between Design A and the original design, which can only reduce the maximum temperature of the piston by 0.22 °C. The position of the cooling gallery of Design C is far from the ring area and closer to the bottom of the piston chamber of the piston, which increases the wall thickness of the ring groove area, reduces the temperature gradient in this area, and reduces the maximum thermal stress of the piston by 2.67 MPa. The position of the cooling gallery in design C is far away from the throat area of piston, which increases the maximum temperature of the piston by 1.15 °C.

**Table 7:** Variable values and results of the clustered designs and the original design.

Design	Variable		SVR		Co-simulation	
	*d*_*1*_ (mm)	*d*_*1*_ (mm)	T (°C)	S (MPa)	T (°C)	S (MPa)
A	3.37	2.64	358.06	74.43	364.28	68.95
B	3.32	4.14	262.11	69.77	365.34	70.52
C	6.14	5.35	366.11	66.50	366.71	68.35
Original	4.10	4.43	362.60	72.91	365.56	71.02

**Figure 12:**
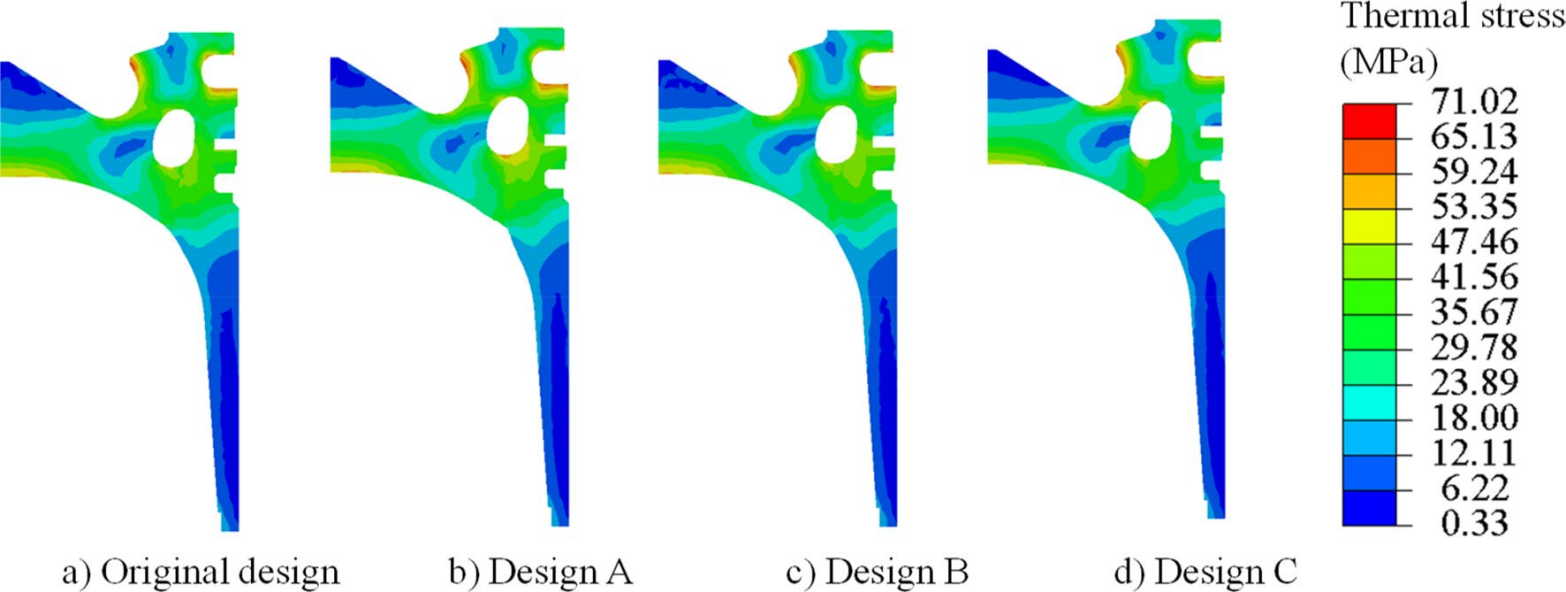
Thermal stress comparison of original design and cluster designs.

The design variables of design A, B, and C are brought into the established fluid–solid coupling heat transfer simulation model of the piston and the cooling gallery for the simulation calculation, and the temperature field and thermal stress distribution cloud diagrams of three design pistons are obtained, as shown in Figs. 11 and 12. It can be seen that the highest temperature of the original design appears in the throat area of piston, and the distribution of temperature field of the piston after optimization is almost unchanged in Fig. 11. It can be seen that the optimized cooling gallery is far away from the areas of piston ring, which makes the thermal stress in the ring area decreased in Fig. 12.

**Figure 13:**
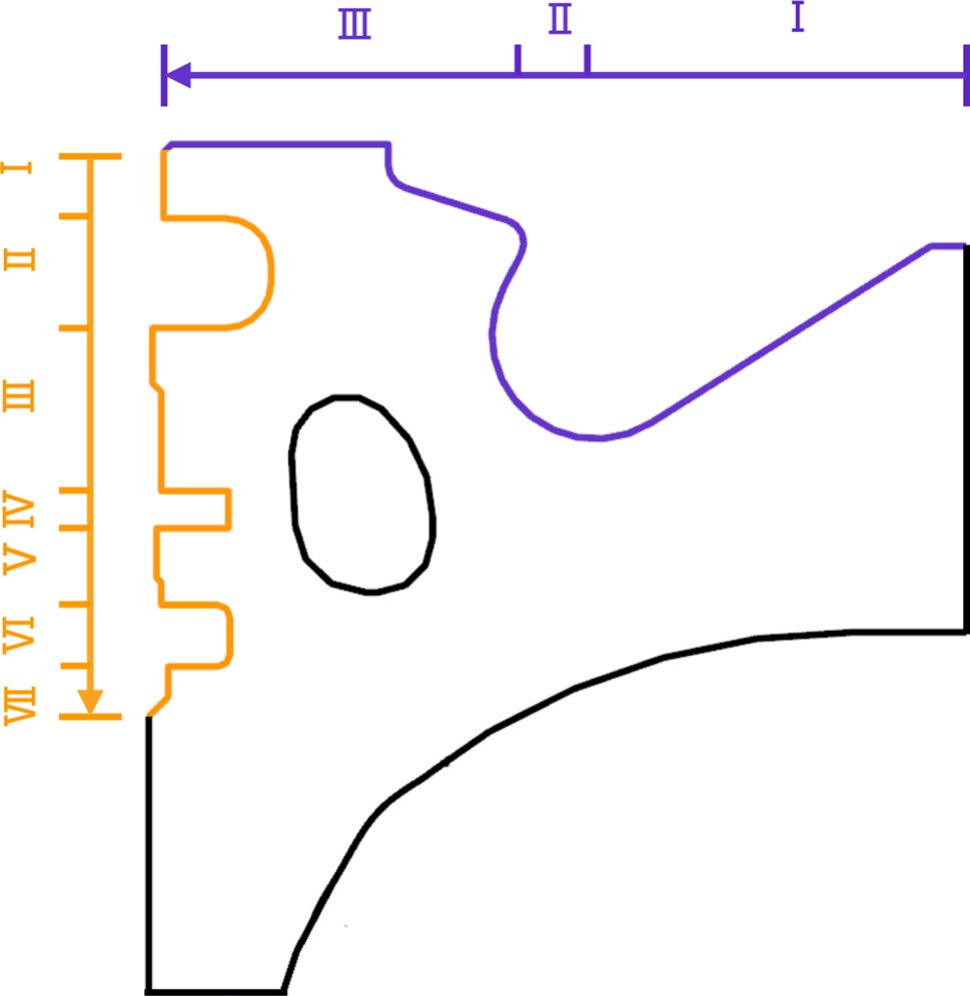
Location of the 50 measuring points.

**Figure 14:**
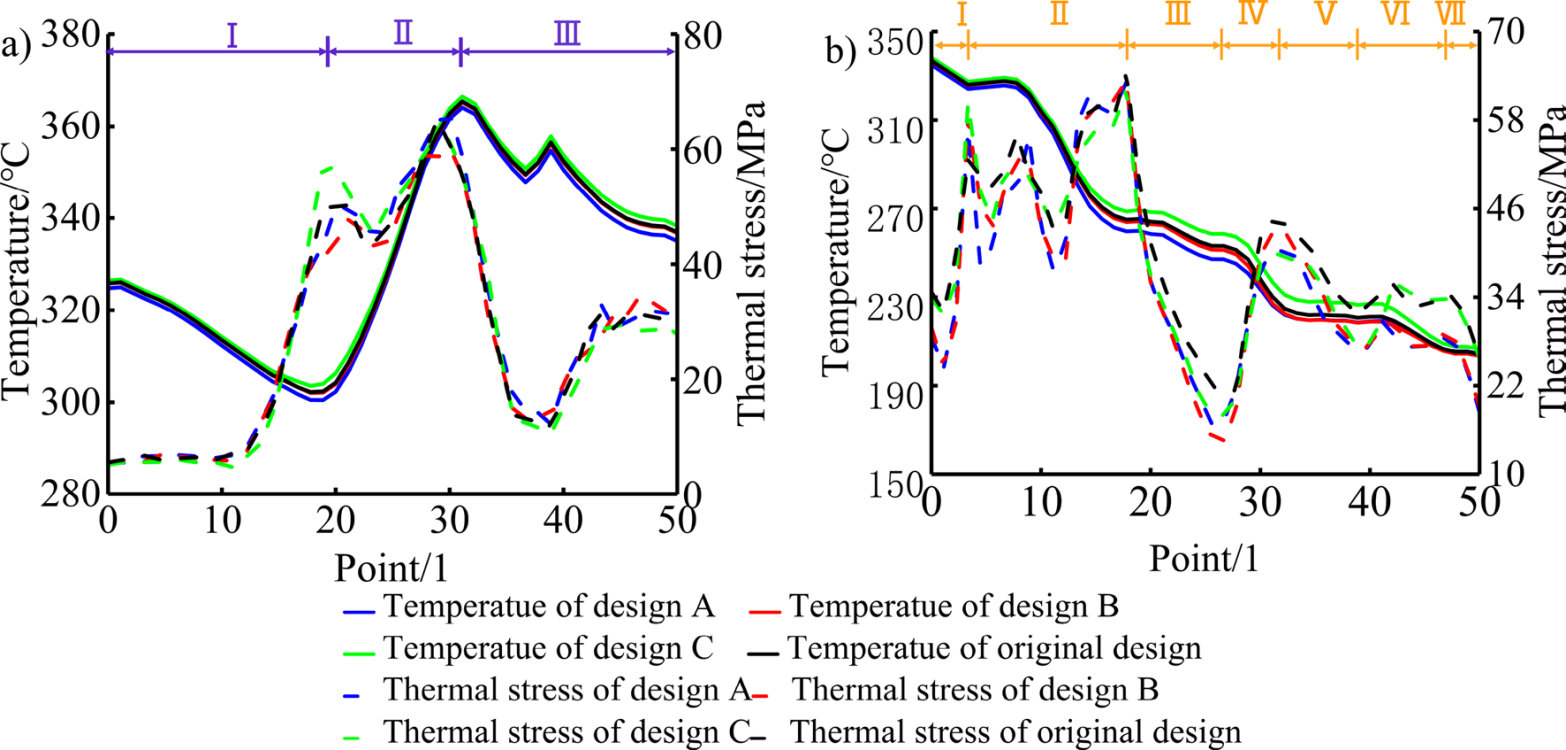
Temperature and thermal stress distribution of the (**a**) top surface and (**b**) piston ring areas.

In order to describe the temperature and thermal stress distribution on the top surface of the piston in more detail, 50 measuring points are selected from the centre of the piston chamber along the direction of the vertical pin hole to the edge of the top surface of the piston for research and analysis, the position is shown in the purple curve in Fig. 13 and the result is shown in Fig. 14a. The temperature distribution on the top surface of the piston of each design is basically the same. The piston reaches the lowest temperature at the bottom of the piston chamber and the highest temperature at the piston throat. Compared with the original design, Design A is farther away from the bottom of the piston chamber and close to the throat, which can transfer the heat of the high-temperature gas in the cylinder to the inner cavity of the piston through the cooling oil cavity, thereby reducing the overall temperature of the piston chamber. Therefore, the temperature at the bottom of the piston chamber is 300.49 °C, which is 1.75 °C lower than the original design 302.24 °C, and the throat temperature is 364.07 °C, which is 1.36 °C lower than the original design 365.43 °C. Design C is closer to the bottom of the piston chamber and away from the throat. Therefore, the temperature at the bottom of the piston chamber is 303.58 °C, which is 1.34 °C higher than the original design, and the temperature at the throat is 366.51 °C, which is 1.08 °C higher than the original design. The change trend of thermal stress on the top surface of the piston of each design is basically the same. Since the bottom area of the piston chamber is at a small distance from the top of the piston inner cavity and the cooling gallery, it can be fully cooled, so that the temperature of the bottom area of the piston chamber drops sharply, resulting in a large temperature difference, resulting in thermal stress concentration. The thermal stress gradually decreases along the wall of the ω-shaped combustion chamber, and the thermal stress rises to the maximum in the region of geometric abrupt change close to the piston throat. In the bottom area of the piston chamber, design B is farther away from this area and has the thickest wall thickness, reducing the temperature gradient in this area and resulting in less thermal stress. The maximum thermal stress is 47.76 MPa, which is 2.48 MPa lower than the original design 50.24 MPa. However, the position of the cooling gallery of design C is closer to this area, and the bottom area of the piston chamber will generate a larger thermal stress, and the maximum thermal stress is 57.17 MPa, which is 6.93 MPa higher than the original design. In the vicinity of the piston throat area, design A is closer to the geometric abrupt area of the throat, resulting in the maximum thermal stress of 66.36 MPa, which is an increase of 1.27 MPa compared to the original design of 65.09 MPa. Design B is farther away from this area, and the maximum thermal stress is 60.23 MPa, which is 4.86 MPa lower than the original design.

In order to describe the temperature and thermal stress distribution in the piston ring area in more detail, 50 measuring points are selected in sequence from the top land along the direction of the vertical pin hole to the top of the piston skirt for research and analysis, the position is shown in the orange curve in Fig. 13 and the result is shown in Fig. 14b. The temperature distribution of the ring area of each design is basically the same, and the temperature decreases step by step from the top of the first land to the top of the skirt. As the cooling gallery of each design is far away from the first ring groove, the temperature gradually increases. Design A is closer to the first ring groove area, with a maximum drop of 6.27 °C compared to the original design. Design C is farther away from the ring groove area, and the maximum increase is 6.75 °C compared to the original design. The variation trend of the thermal stress in the piston ring area of each design is basically the same. At the position where the geometric structure of piston ring groove changes suddenly, the thermal stress has a trough peak. The design thermal stress on the lower side of the first ring groove reaches the maximum. The location of the cooling gallery in Design A and Design B, although closer to the piston ring area and with a smaller wall thickness than the original solution, is closer to the throat area where the highest temperatures are generated, allowing more heat to be carried away and reducing the temperature gradient in this area. While Design C has the thickest wall thickness in this area, resulting in a lower temperature gradient in this area. Therefore, the maximum thermal stresses of design A, B, and C are all smaller than the original design, which is 0.35 MPa, 0.07 MPa, and 2.42 MPa respectively lower than the original design of 63.54 MPa. The minimum thermal stress of each design appears in the second land. The minimum thermal stresses of the optimized designs A, B, and C are also smaller than the original design, which is 0.94 MPa, 3.19 MPa, and 8.43 MPa lower than the original design of 17.82 MPa, respectively.

For the maximum temperature of the piston, by reducing the distance between the cooling gallery and the throat area, the heat of the high-temperature gas in the cylinder can be transferred to the inner cavity of the piston via the cooling gallery, thereby reducing the maximum temperature of the piston. For the maximum thermal stress of the piston, the temperature gradient of the ring area can be reduced by reducing the distance between the cooling gallery and the throat area or increasing the distance between the cooling gallery and the ring area, thereby reducing the maximum thermal stress of the piston.

### Analysis of heat transfer characteristics of piston under transient conditions

**Figure 15:**
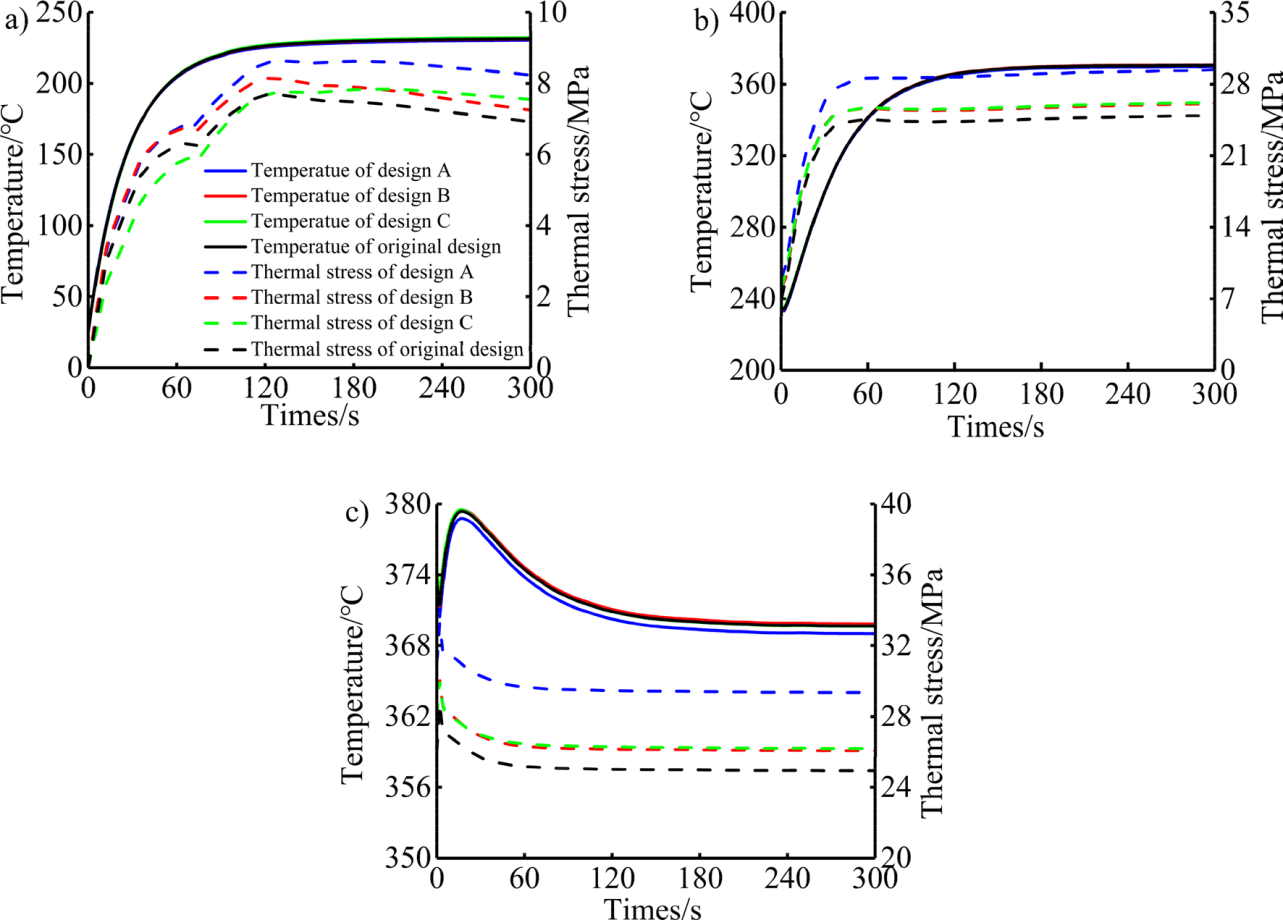
Temperature and thermal stress distribution of throat under the (**a**) cold start condition, (**b**) urgent acceleration condition and (**c**) rapid deceleration condition.

**Figure 16:**
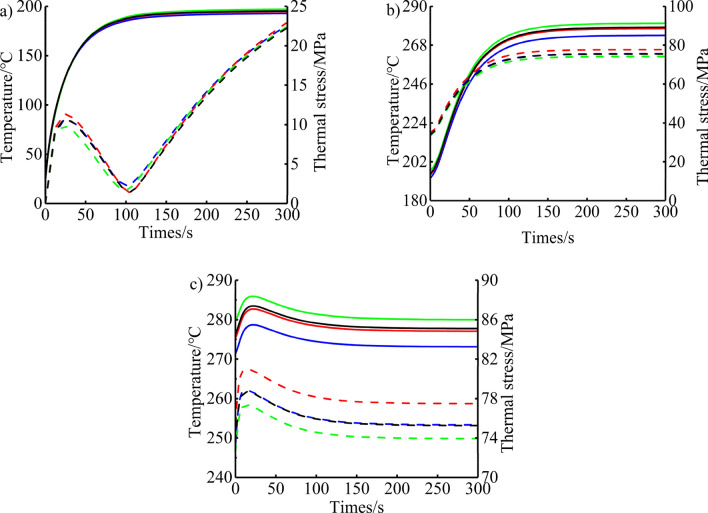
Temperature and thermal stress distribution of the first ring groove under the (**a**) cold start condition, (**b**) urgent acceleration condition and (**c**) rapid deceleration condition.

According to the analysis of the maximum torque condition, the maximum temperature of the piston appears in the throat area, and the maximum thermal stress appears in the first ring groove area. In order to analyse the transient changes of the temperature and thermal stress of the piston throat and the first ring groove in the transitional conditions in more detail, as shown in Figs. 15 and 16. The temperature changes of the piston throat and the first ring groove tend to be consistent. Before the cold start, the piston temperature is the same as the ambient temperature. After the cold start, it is affected by the sudden addition of high-temperature heat source on the top surface of the piston, forming a large temperature gradient, and the convective heat transfer coefficient increased rapidly, resulting in an exponential upward trend of temperature within the first 80 s of the cold start. As the cold start continues, the heat is gradually transferred from the piston head to the piston skirt due to the thermal conduction of the piston material, and the gradient of temperature gradually decreases. The heat transfer rate becomes slow and tends to be stable, the temperature rise gradually becomes flat, and the temperature does not change after 100 s. During the process of cold start, the maximum change in the temperature of the piston throat and the first ring groove reached 207.29 °C and 172.00 °C, respectively. After the process of urgent acceleration started, the fuel injection amount should be increased when the engine rapidly increases the speed and load from the idle speed condition to the calibrated speed condition. This leads to the increase of heat load in the cylinder, the rapid increase of gas temperature, and the rapid increase of convective heat transfer coefficient on the top surface of the piston, which makes the piston temperature show an exponential upward trend in the first 100 s. Subsequently, the temperature rise gradually became flat, and the temperature no longer changed after 130 s. During the urgent acceleration process, the maximum change in the temperature of the piston throat and the first ring groove reached 136.78 °C and 83.52 °C, respectively. After the rapid deceleration process started, the engine condition decelerates sharply from the steady state to the maximum torque condition. The overall thermal load of the piston does not change much, and it is affected by the thermal shock of the stable cycle of the engine, which causes the temperature of the top surface of the piston to rise briefly in the early stage of the rapid deceleration process, reaching the maximum value in about 20 s. Then the temperature decreased slowly and slightly, and reached stability in 200 s. During the rapid deceleration process, the maximum change in the temperature of the piston throat and the first ring groove reached 9.89 °C and 7.36 °C, respectively.

Under cold start conditions, due to the thermal inertia of the piston material, a large temperature gradient is formed in the piston in the early stage of the cold start, so that the thermal stress of the piston throat increases rapidly in the first 70 s. Then the thermal stress continued to rise at a rate lower than the previous rise and reached its maximum at 120 s. Subsequently, due to the thermal conduction of the piston, the temperature gradient of the piston decreases gradually, resulting in a gradual decrease in thermal stress. Under the condition of urgent acceleration, the thermal stress of the piston throat shows a rapid increase first, and gradually tends to a stable change after 100 s. Under the condition of rapid deceleration, the thermal stress of the piston throat first increased rapidly, then decreased, and then increased again until it reached a stable trend. The maximum change in thermal stress of the piston throat during cold start, urgent acceleration, and rapid deceleration are 8.62 MPa, 20.43 MPa, and 4.08 MPa, respectively. Among them, design A, because the cooling gallery is closer to the piston throat area, rises by 0.93 MPa, 4.44 MPa, and 4.53 MPa, respectively, compared to 7.69 MPa, 24.99 MPa, and 28.74 MPa at the end of the original design cold start condition, urgent acceleration condition, and rapid deceleration condition. The thermal stress of the first ring groove of the piston increases rapidly during the process of cold start, reaches a small peak at 25 s, then decreases, then increases again and reaches the maximum of thermal stress at 300 s. The reason why the thermal stress increases again is that the piston is continuously heated by the relatively stable high temperature gas in the cylinder after the rotational speed is stabilized at the idle speed condition, and the temperature field of the piston gradually tends to the steady state under the idle speed condition, which makes the thermal stress gradually increase. The maximum change in thermal stress is 22.96 MPa. The thermal stress change law of the first ring groove of the piston under urgent acceleration and rapid deceleration is the same as that at the throat, and the maximum changes are 43.10 MPa and 5.72 MPa, respectively. Design B is closer to the first ring groove of the piston, and after stabilization, it increases by 2.23 MPa and 2.28 MPa respectively compared to the original design at 75.57 MPa for urgent acceleration and 78.70 MPa for rapid deceleration. And design C is more away from the first ring groove of the piston, and after stabilization, it is 1.33 MPa and 1.45 MPa lower respectively compared to the original design.

## Conclusions

In this paper, the sequential coupling method combined with the transient temperature test under the transitional conditions of the piston is used to establish a simulation model of the fluid–solid coupling heat transfer between the piston and the cooling gallery. Through this simulation model, the temperature field and thermal stress of the piston under the different position of cooling gallery can be accurately obtained. The Pareto optimization algorithm is introduced to optimize the position of the cooling gallery to reduce the maximum temperature and maximum thermal stress of the piston, and extract three typical solutions of the optimal solution concentration for heat transfer simulation calculation. Considering these factors, the following conclusions are drawn.

1. There are 3 typical solutions extracted by the algorithm of k-means clustering. Compared with the original design, the maximum temperature of Design A decreases by 1.28 °C while the maximum thermal stress decreases by 2.07 MPa. The maximum temperature and maximum thermal stress of Design B decrease by 0.22 °C and 0.5 MPa, respectively. The maximum thermal stress of Design C decreases by 2.67 MPa when the maximum temperature increases by 1.15 °C. The design of 3 typical solutions shows that the location of the cooling gallery should be close to the throat area and away from the ring area.
2. The temperature field and thermal stresses of the piston throat and first ring groove of the three typical designs and the original design change dramatically during the cold start with maximum changes reaching 207.29 °C and 8.62 MPa for the throat and 172.00 °C and 22.96 MPa for the first ring groove respectively. In the early stages of cold start, due to the sudden high-temperature heat source on the top surface of the piston, resulting in an exponential rise in the temperature of the piston throat and the first ring groove, both of which reach a stable temperature after 100 s. However, the thermal stress trends of the two are not the same during the cold start. The reason why the thermal stress at the first ring groove increases again is that the piston is continuously heated by the relatively stable high temperature gas in the cylinder after the idle speed condition, and the temperature field of the piston gradually tends to the steady state.
3. The temperature field and thermal stresses of the piston throat and first ring groove of the three typical designs and the original design change dramatically during urgent acceleration and the trends are consistent, with the maximum changes reaching 136.78 °C and 20.43 MPa for the throat and 83.52 °C and 43.10 MPa for the first ring groove respectively. In the early stage of urgent acceleration, the temperature and thermal stress of the piston throat and the first ring groove increased exponentially due to the increase of fuel injection. Subsequently, due to the thermal conduction of the piston material, the temperature and thermal stress of the piston throat and the first ring groove gradually tend to be stable.
4. The laws of the temperature field and thermal stress of the piston throat and first ring groove of the three typical designs and the original design are consistent during the rapid acceleration, with the maximum changes reaching 9.89 °C and 4.08 MPa for the throat and 7.36 °C and 5.72 MPa for the first ring groove respectively. In the early stage of rapid deceleration, the temperature and thermal stress of the piston throat and the first ring groove temporarily increased to the maximum value due to the effect of the thermal shock of the stable cycle of the engine. Subsequently, the temperature and thermal stress of the piston throat and the first ring groove gradually tend to be stable.

## Data Availability

The raw data required to reproduce these findings cannot be shared at this time as the data also forms part of an ongoing study.

## List of symbols

*d*_*1*_: Structural variable (mm)
*d*_*2*_: Structural variable (mm)
*e*: Constant
*f*_*1*_: Maximum thermal stress (MPa)
*f*_*2*_: Maximum temperature (°C)
*S*: Thermal stress (MPa)
*T*: Temperature (°C)

## Greek symbols

*Ɛ*: Insensitive loss function

## Subscripts

*∆*: Increment
